# Mean Platelet Volume in Patients with Nonarteritic Anterior Ischemic Optic Neuropathy

**DOI:** 10.1155/2016/1051572

**Published:** 2016-02-04

**Authors:** Muhammed Şahin, Alparslan Şahin, Bilal Elbey, Harun Yüksel, Fatih Mehmet Türkcü, Abdullah Kürşat Cingü

**Affiliations:** ^1^School of Medicine, Department of Ophthalmology, Dicle University, 21280 Diyarbakir, Turkey; ^2^School of Medicine, Department of Immunology, Dicle University, 21280 Diyarbakir, Turkey

## Abstract

*Objective*. We aimed to investigate the mean platelet volume (MPV) of the patients with nonarteritic anterior ischemic optic neuropathy (NAION).* Methods*. The medical records of 46 patients with the diagnosis of NAION and 90 control subjects were retrospectively evaluated. All participants underwent complete ocular examination including intraocular pressure (IOP) measurement. Hematocrit, MPV, hemoglobin, and platelet levels of the patients with NAION were compared with those of control subjects.* Results*. There was no significant difference between the groups in platelet counts (*p* = 0.76). NAION group had significantly higher MPV values (8.25 ± 1.26 fL) than that of control subjects (7.64 ± 1.01 fL) (*p* < 0.001). Multivariate logistic regression analysis showed that MPV is an independent predictor of NAION (odds ratio = 1.61; 95% confidence interval (CI) = 1.13–2.28; *p* = 0.007). The mean IOP was significantly higher in NAION group (*p* < 0.001). IOP was also found as an independent predictor of NAION according to the regression analysis (OR = 1.27; 95% CI = 1.08–1.48; *p* = 0.003).* Conclusion*. Our results demonstrated that the MPV values were significantly higher in NAION patients, suggesting that larger platelets may contribute to the pathogenesis of the NAION.

## 1. Introduction

Anterior ischemic optic neuropathy (AION) is a condition with unilateral, painless, and sudden visual loss caused by acute ischemic damage of the anterior region of the optic nerve in elderly patients. AION results in a vascular insufficiency in the short posterior ciliary arteries (SPCA). There are two types of AION: arteritic (AAION) and nonarteritic (NAION) [1–3]. As its most common form, NAION is a multifactorial disease. Although the pathogenesis of NAION is somewhat known, it is still highly complex to understand completely and several potential risk factors such as diabetes mellitus (DM), arteriosclerosis, crowded disc, hypertension (HT), ischemic heart disease, prothrombotic states, carotid disease including occlusion and dissection, emboli, and nocturnal hypercoagulability in sleep apnea syndrome have been demonstrated [2, 4–9]. Moreover, thrombophilia, increased fibrinogen or cholesterol levels, and von Willebrand factor antigen have been shown to be of significant importance in the development of NAION [2, 10]. On the other hand, it has been reported that glucose-6-phosphate dehydrogenase (G6PDH) deficiency has a protective effect against the development of NAION and retinal vein occlusion (RVO) [11, 12]. Pinna et al. [12] explained this protection with the reduced ability of esterification and accumulation of cholesterol in the arteries G6PDH deficient subjects.

Platelets have great importance in the pathogenesis of vasoocclusive diseases. Large platelets are more reactive than small platelets and produce more thromboxane A_2_ and express more glycoprotein Ib and glycoprotein IIb/IIIa receptors on their surfaces resulting in an increased propensity to aggregate. Mean platelet volume (MPV) is an indicator of platelet size, function, and activation [13, 14]. Multiple reports have investigated MPV values in patients with Behçet's disease, rheumatoid arthritis, myocardial infarction, DM, HT, pseudoexfoliation syndrome, and RVO [15–19].

In the present study, we aimed to investigate if the platelets were involved in the development of NAION through increased MPV. To the best of our knowledge, it is the first report evaluating the MPV levels in patients with NAION.

## 2. Methods

The study protocol was approved by the local ethic committee and the study was conducted in accordance with the Declaration of Helsinki. The patients who were diagnosed as NAION between January 2011 and April 2015 were reviewed retrospectively. All subjects underwent a full ocular examination including measurement of visual acuity (VA) with Snellen chart and intraocular pressure (IOP), slit lamp biomicroscopic anterior segment, and fundus examination. Snellen VA were converted to logarithm of the minimal angle of resolution (logMAR) for statistical analysis.

Age, gender, ocular pathology, systemic disease, and complete blood count (CBC) parameters (i.e., MPV, hemoglobin (Hb), hematocrit (Hct), and platelet count) were recorded. NAION was diagnosed with presence of the following: impaired vision, relative afferent pupillary defect, visual-field defects consistent with optic neuropathy, and characteristic fundus changes (swollen and pale peripapillary flame-shaped hemorrhage). The participants who had any systemic disease other than HT and DM were excluded from the study. Patients with anemia (Hct below 38.0%), any cardiovascular disease, chronic renal failure, history of statin use, atrial fibrillation, inflammatory diseases such as rheumatoid arthritis, history of smoking or alcohol consumption, glaucoma, and/or a history of any ocular surgery or trauma were also excluded [20]. Age and gender matched subjects in the control group were recruited from outpatient clinic of ophthalmology with minor refractive errors (±1.0 diopter).

Blood samples were taken at the time of diagnosis of NAION. CBC samples, which were drawn into vacutainer tubes containing 0.04 mL of the 7.5% K3 salt of EDTA, were analyzed within an hour after sampling using a commercially available analyzer (CELL-DYN 3700, Abbott Diagnostics, Abbott Park, IL, USA). Platelet count, MPV, Hb, erythrocyte sedimentation rate, c-reactive protein, and Hct parameters of the subjects were recorded. Normal MPV values ranged between 7.0 and 10.4 fL.

### 2.1. Statistical Analysis

All values were given as mean ± SD. Statistics was performed using SPSS versus 11.5 statistical package program. Categorical variables were compared with the* chi-square* test. The* Kolmogorov-Smirnov* test was applied to test the distribution pattern of each data between two groups. The* Student's t-test* was used for normally distributed data and the* Mann-Whitney U* test was used for not normally distributed data in group comparisons. A *p* value less than 0.05 was considered significant. Multivariate logistic regression analysis was used to assess the associations among MPV, Hct and Hb levels, IOP, HT, DM, age, and gender with NAION.

## 3. Results

Patients with NAION were consecutively recruited at the Dicle University, School of Medicine, Department of Ophthalmology. A total of 46 patients with NAION were eligible for the study. Control group consisted of 90 subjects. The mean age of the NAION patients and the control subjects was 57.3 ± 9.1 and 55.7 ± 13.2 years, respectively. Male-to-female ratio was 23/23 in the NAION group and 39/51 in the control group. There were no statistical differences in age and sex between groups (*p* = 0.2, *p* = 0.1, resp.) (Table 1). There was no significant difference between control and NAION groups with respect to presence of HT and DM (*p* = 0.5). There was no difference between groups regarding anti-HT and antidiabetic drug use.

The mean MPV was significantly higher in patients with NAION than that of control group (8.25 ± 1.26 fL versus 7.64 ± 1.01 fL, *p* < 0.001). The mean platelet count was similar in the control and NAION groups, 264.3 ± 57.8 × 10^3^/*μ*L and 263.7 ± 67.5 × 10^3^/*μ*L, respectively (*p* = 0.76). Multivariate logistic regression analysis showed that MPV was also an independent predictor of NAION (odds ratio (OR) = 1.61; 95% confidence interval (CI) = 1.13–2.28; *p* = 0.007). The mean IOP was significantly higher in NAION group than that of control group (*p* < 0.001). Multivariate logistic regression analysis showed that IOP was also an independent predictor of NAION (OR = 1.27; 95% CI = 1.08–1.48; *p* = 0.003).

## 4. Discussion

The present study showed increased levels of MPV in patients with NAION. To the best of our knowledge, this is the first paper that shows the relationship between increased MPV values in patients with NAION.

Nonarteritic anterior ischemic optic neuropathy is the second most common optic neuropathy in elderly patients after glaucomatous optic nerve damage, which is the typical NAION [21]. The etiology of NAION is multifactorial with systemic hemodynamic disorders and local anatomical factors, both playing a role [8, 9, 22, 23]. In NAION, the perfusion of the SPCA is damaged and infarction of the optic nerve head develops. Macro and micro vascular disorders play key role in the pathogenesis of reduced perfusion [4]. At the same time, atherosclerosis accompanying HT, DM, and ischemic heart disease also plays an important role in the pathogenesis of NAION.

Several large series have reported that 10.5% to 15% of NAION patients are younger than 45 years [22, 24, 25]. Vascular rheological microcirculatory impairment is the basis of the pathogenesis in young NAION (yNAION). According to the previous studies, HT and DM were associated strongly with the development of yNAION [22, 24, 25]. Moreover, hypercholesterolemia may be the first manifestation of elevated serum lipids in yNAION [26]. Recently, Pinna et al. [12] reported the protective effect of the G6PDH deficiency on the progression of the atherosclerotic process. As a result they suggested that the reduced ability to esterify and accumulate cholesterol in the arteries may account for a lower risk for atherosclerotic disease, as well as NAION in G6PDH deficient subjects.

The haemostatic balance is maintained by complex interactions between the platelets, vessel wall, the coagulation system, physiological anticoagulants, and the fibrinolytic system. Platelet hyperactivity may also contribute to hypercoagulability in patients with NAION. Salomon et al. [4] investigated the pathogenic role of different risk factors of thrombophilia but found no significant differences in NAION patients. In addition, Talks et al. [27] suggested that hypercholesterolemia and high fibrinogen levels may have a role in the development of NAION. Besides, Nagy et al. [10] suggested that thrombophilia due to factor V Leiden mutation seems to play an important role in the development of microthrombi in NAION.

The platelets have also an important role in the pathogenesis of thromboocclusive diseases. Since larger platelets store and release larger amounts of serotonin and *β*-thromboglobulin and produce more thromboxane A_2_, they are more reactive and prone to aggregation [28, 29]. MPV is the indicator of the activity and the size of platelets. Increased values of MPV have been reported to be a risk factor for deep venous thrombosis, stroke, acute ischemic cerebrovascular events, and acute myocardial infarction [28–33].

The platelet functions in NAION have been studied. Hayreh suggested that serotonin released by platelet aggregation in atherosclerotic plaques may play an important role in the pathogenesis of NAION, by producing transient nonperfusion or hypoperfusion of the optic nerve head [34]. Nagy et al. [26] reported that the platelet P-selectin level was increased in patients with NAION. They suggested that enlarged platelet activity may be related to NAION. Levels of P-selectin may increase in different diseases with enhanced platelet reactivity and accompanied vessel occlusion, such as DM, arteriosclerosis, stroke, and peripheral artery disease [26]. Besides, Salomon et al. [5] examined the role of platelet glycoprotein polymorphism in NAION. This study demonstrated that increased platelet aggregation owing to the presence of the polymorphism can have a predisposing influence in the pathophysiology of NAION.

As larger platelets are hemostatically more active, they produce more prothrombotic factors [35]. Bath and Butterworth reported that the platelet hyperactivity results in an increase in MPV [36]. Larger platelets aggregate easier than smaller ones [29]. In our study, the MPV values were significantly higher compared to control group which may contribute to the pathogenesis of NAION. In our study, multivariate logistic regression analysis revealed that MPV is an independent predictor for NAION. The presence of high MPV in these patients may increase the risk of NAION. The mean MPV value is controversial. In a recently published MPV study from Turkey the mean MPV of healthy individuals was 8.9 ± 1.4 fL [37]. They have evaluated the MPV measurement within the first 6 hours. However, MPV increases over time as platelets swell in EDTA; therefore optimal MPV measurement should be within 2 hours of venipuncture [38]. Contrary in a study from Italy the mean MPV of outpatients was found to be 7.7 fL (6.4–9.8 fL) [39].

In the present study, the mean IOP was significantly higher in NAION patients compared to control subjects. Although our results demonstrated that the IOP is an independent predictor in NAION, its effect on NAION is still controversial. Rothová and Boguszaková [40] reported an increase of IOP in 4 of 17 NAION patients. Contrary Kalenak et al. [41] did not report association between NAION and IOP (16.3 versus 16.1 mm Hg). Although the mean IOP values in our study were significantly higher in NAION patients, it has no clinical importance because the measurements were within the normal range (15.8 versus 14.1 mm Hg). Moreover lack of central corneal thickness (CCT) and cup/disc (c/d) ratio was the limitations of such a comparison with respect to IOP in the present study.

Mean platelet volume has been studied in a few ocular vascular disorders. Our team reported an increase of the MPV in patients with RVO and ocular Behçet's disease [3, 6]. Ateş et al. [42] found a significant increase of the MPV in patients with diabetic retinopathy (DR). They reported a correlation between the severity of DR and MPV values.

Our study has some limitations: it was a retrospective analysis with a relatively small number of patients, which is inherent to investigations of multifactorial diseases. With a lack of body mass index, lipid profile of the patients and history of metabolic syndrome [43], CCT, c/d ratio, and the results demonstrate only the hematologic status at the acute stage of NAION. Thus, these results may not reflect the status of these patients over long periods. Because of the low incidence of NAION, an adequately controlled prospective study would be difficult but worthy of further workup.

## 5. Conclusion

Mean platelet volume was significantly higher in patients with NAION. Despite the retrospective nature of our study, we suggest that MPV may be used as a predictive tool for identifying risk of NAION in the future. However, increased MPV alone is not responsible for NAION as its pathogenesis is more complex and multifactorial. Further studies are needed to confirm the predictive value of MPV for NAION risk.

## Figures and Tables

**Table 1:** The demographic and clinical features of the NAION and control groups.

	NAION	Control	*p*	Power estimated (%)
Age (year)	57.3 ± 9.1	55.7 ± 13.2	0.2^c^	
Gender (M/F)	23/23	39/51	0.1^a^	
HT	6/46	8/90	0.5^a^	
DM	6/46	16/90	0.5^a^	
HT and DM	7/46	15/90	0.5^a^	
Hb (g/dL)	14.1 ± 1.4	13.8 ± 1.3	0.2^c^	33.2
Hct (%)	42.4 ± 4	41.3 ± 3	0.2^c^	
Plt (10^3^/*µ*L)	263.7 ± 67.5	264.3 ± 57.8	0.76^c^	5.6
MPV (fL)	8.25 ± 1.26	7.64 ± 1.01	**<**0.001^b^	**88.6**
IOP (mm Hg)	15.8 ± 3.4	14.1 ± 2.0	**<**0.001^b^	**93.1**
VA (LogMAR)	1.19 ± 0.94	0.09 ± 0.2	**<**0.001^b^	
CRP (mg/L)	0.46 ± 0.4	0.50 ± 0.3	0.78^c^	
ESR (mm/hour)	11.8 ± 7.7	12.0 ± 8.8	0.88^b^	

NAION: nonarteritic anterior ischemic optic neuropathy, HT: hypertension, DM: diabetes mellitus, Hb: hemoglobin, Hct: hematocrit, Plt: platelet count, MPV: mean platelet volume, IOP: intraocular pressure, VA: visual acuity, CRP: c-reactive protein, and ESR: erythrocyte sedimentation rate.

^a^Chi-square test

^b^Mann-Whitney *U* test

^c^ *t*-test.
